# DNA Methylation in Inflammatory Pathways Modifies the Association between BMI and Adult-Onset Non-Atopic Asthma

**DOI:** 10.3390/ijerph16040600

**Published:** 2019-02-19

**Authors:** Ayoung Jeong, Medea Imboden, Akram Ghantous, Alexei Novoloaca, Anne-Elie Carsin, Manolis Kogevinas, Christian Schindler, Gianfranco Lovison, Zdenko Herceg, Cyrille Cuenin, Roel Vermeulen, Deborah Jarvis, André F. S. Amaral, Florian Kronenberg, Paolo Vineis, Nicole Probst-Hensch

**Affiliations:** 1 Swiss Tropical and Public Health Institute, 4051 Basel, Switzerland; a.jeong@swisstph.ch (A.J.); medea.imboden@swisstph.ch (M.I.); christian.schindler@swisstph.ch (C.S.); 2 Department of Public Health, University of Basel, 4001 Basel, Switzerland; 3 International Agency for Research on Cancer, 69372 Lyon, France; ghantousa@iarc.fr (A.G.); NovoloacaA@students.iarc.fr (A.N.); hercegz@iarc.fr (Z.H.); CueninC@iarc.fr (C.C.); 4 ISGlobal, Barcelona Institute for Global Health, 08003 Barcelona, Spain; anneelie.carsin@isglobal.org (A.-E.C.); manolis.kogevinas@isglobal.org (M.K.); 5 Universitat Pompeu Fabra (UPF), 08002 Barcelona, Spain; 6 CIBER Epidemiología y Salud Pública (CIBERESP), 08005 Barcelona, Spain; 7 Department of Economics, Business and Statistics, University of Palermo, 90128 Palermo, Italy; gianfranco.lovison@unipa.it; 8 Environmental Epidemiology Division, Utrecht University, Institute for Risk Assessment Sciences, 3584CM Utrecht, The Netherlands; R.C.H.Vermeulen@uu.nl; 9 Population Health and Occupational Disease, National Heart and Lung Institute, Imperial College, London SW3 6LR, UK; d.jarvis@imperial.ac.uk (D.J.); a.amaral@imperial.ac.uk (A.F.S.A.); 10 Division of Genetic Epidemiology, Medical University of Innsbruck, 6020 Innsbruck, Austria; Florian.Kronenberg@i-med.ac.at; 11 MRC-PHE Centre for Environment and Health, School of Public Health, Imperial College London, London W2 1PG, UK; p.vineis@imperial.ac.uk; 12 Italian Institute for Genomic Medicine (IIGM), 10126 Turin, Italy

**Keywords:** adult-onset asthma, non-atopic asthma, obesity, inflammation, innate immunity, epigenetics, DNA methylation, epigenome-wide association study

## Abstract

A high body mass (BMI) index has repeatedly been associated with non-atopic asthma, but the biological mechanism linking obesity to asthma is still poorly understood. We aimed to test the hypothesis that inflammation and/or innate immunity plays a role in the obesity-asthma link. DNA methylome was measured in blood samples of 61 non-atopic participants with asthma and 146 non-atopic participants without asthma (non-smokers for at least 10 years) taking part in the Swiss Cohort Study on Air Pollution and Lung and Heart Diseases in Adults (SAPALDIA) study. Modification by DNA methylation of the association of BMI or BMI change over 10 years with adult-onset asthma was examined at each CpG site and differentially methylated region. Pathway enrichment tests were conducted for genes in a priori curated inflammatory pathways and the NLRP3-IL1B-IL17 axis. The latter was chosen on the basis of previous work in mice. Inflammatory pathways including glucocorticoid/PPAR signaling (*p* = 0.0023), MAPK signaling (*p* = 0.013), NF-κB signaling (*p* = 0.031), and PI3K/AKT signaling (*p* = 0.031) were enriched for the effect modification of BMI, while NLRP3-IL1B-IL17 axis was enriched for the effect modification of BMI change over 10 years (*p* = 0.046). DNA methylation measured in peripheral blood is consistent with inflammation as a link between BMI and adult-onset asthma and with the NLRP3-IL1B-IL17 axis as a link between BMI change over 10 years and adult-onset asthma in non-atopic participants.

## 1. Introduction

Obesity and overweight have repeatedly been linked to asthma [1,2], with several studies reporting a stronger association of obesity or overweight with non-atopic compared to atopic asthma [3,4] and with late-onset asthma compared to early-onset asthma [5,6]. We previously observed heterogeneity of the overweight-asthma association across asthma classes identified by latent class analysis [7].

The biological mechanism linking obesity and overweight with asthma is poorly understood. Excessive adipose tissue may increase the work associated with breathing, reduce lung volume, and promote airway hyperresponsiveness and airway narrowing [8,9]. However, the more likely hypothesis is that this association is not entirely mechanical, but that chronic inflammation related to overweight and obesity contributes to asthma development. Adiposity is characterized by dysregulated production of pro-inflammatory cytokines and infiltration and activation of macrophages [10,11]. While M2 macrophages are predominant in non-obese adipose tissue, pro-inflammatory M1 macrophages increase in obese adipose tissue, leading to low-grade chronic systemic inflammation [12]. Whether and how obesity and overweight leads to airway inflammation is controversial. An interesting finding in mice experiments pointed to NLRP3 (nucleotide-binding domain, leucine-rich repeats-containing family, pyrin domain-containing-3) inflammasome and interleukin-17 (IL17) producing innate lymphoid cell group 3 (ILC3) cells as a link between obesity and airway hyperresponsiveness (AHR) [13]. Upon recognition of various danger signals, NLRP3 inflammasome produces interleukin-1β (IL1B) via caspase-1. IL1B, in turn, activates ILC3 cells to produce IL17, leading to AHR. Kim and her colleagues demonstrated that the NLRP3-IL1B-IL17 axis is crucial in AHR development in obese mice [13].

High-throughput arrays allow cost-effective genome-wide quantification of DNA methylation. The epigenome-wide association study (EWAS) design has been successfully applied to identify methylation markers measured in peripheral blood related to a variety of endogenous and environmental insults as well as health outcomes. Recently, the largest EWAS on asthma identified DNA methylation at several immunity and inflammation related CpG sites to be associated with asthma in children [14,15,16]. Several studies reported methylation markers of obesity and overweight measured in peripheral blood, which in part reflected inflammatory pathways [17,18,19]. Rastogi and her colleagues reported differential DNA methylation in obese children with non-atopic asthma that was consistent with a role of inflammation [20]. However, differential DNA methylation related to obesity-associated asthma in adults is largely unexplored. An earlier EWAS study in adults showed heterogeneity in differential DNA methylation patterns across inflammatory sub-phenotypes of asthma [21].

In light of the suggestive evidence for inflammation as a mediator in the overweight/obesity-asthma link, we formally explored whether interaction signals between body mass index (BMI) or BMI change and DNA methylation in peripheral blood on non-atopic adult-onset asthma are enriched for signals mapping to inflammatory pathways. Specifically, we tested the hypothesis by conducting an epigenome-wide interaction study (EWIS) followed by candidate pathway enrichment analysis for a priori curated inflammatory pathways and the NLRP3-IL1B-IL17 axis, making use of the information from the Swiss Cohort Study on Air Pollution and Lung and Heart Diseases in Adults (SAPALDIA). Identification of differential DNA methylation enriched in the candidate pathways would add further support that inflammation and/or innate immunity play a role in overweight-asthma link, although the inflammation was not directly measured. This hypothesis-driven approach was corroborated by agnostic pathway enrichment analysis in combination with differentially methylated region (DMR) analysis.

## 2. Materials and Methods

### 2.1. Study Samples

The Swiss Cohort Study on Air Pollution and Lung and Heart Diseases in Adults was initiated in 1991 (SAPALDIA1), recruiting 9651 participants in eight regions representing various meteorological and geographical environments in Switzerland. Of the 9651 participants, 8047 and 6088 were followed-up in the second and the third survey, respectively (SAPALDIA2 in 2001–3 and SAPALDIA3 in 2010–11). The detailed study protocol was reported previously [22,23].

We conducted a nested case-control study of adult-onset asthma among the non-atopic SAPALDIA3 participants, all of whom were non-smokers for at least 10 years before blood draw and interview. Cases were selected among the participants with self-reported asthma and self-reported age of onset later than 16 years based on the availability of archived blood samples and covariate information. Controls were randomly selected among the participants who never reported the following throughout the surveys: self-reported asthma, physician-diagnosed asthma, asthma attack in the last 12 months, current asthma medication, wheezing without cold in the last 12 months, three or more asthma-related symptoms in the last 12 months (symptoms considered: breathless while wheezing, woken up with a feeling of chest tightness, attack of shortness of breath after exercise, attack of shortness of breath while at rest, woken by attack of shortness of breath). Cases and controls with positive skin prick test at baseline defined as an adjusted mean wheal diameter ≥3 mm to at least one of eight common respiratory allergens were excluded (allergens considered: cat fur, dog epithelia, house dust mite (*Dermatophagoides pteronyssinus*), timothy grass pollen, birch pollen, *Parietaria* pollen, and the molds *Alternaria* and *Cladosporium*). In total, 61 cases and 146 controls were examined in the EWIS followed by the pathway enrichment tests. Study samples’ characteristics are summarized in Table 1. All participants gave written informed consent, and ethical approval was obtained from the Swiss Academy of Medical Sciences and the regional committees for each study center.

### 2.2. Covariates

Weight and height were measured, and BMI was computed as weight in kilograms divided by the square of height in meters. BMI change was defined as the difference in BMI between SAPALDIA3 and SAPALDIA2. Negative values of the BMI change mean reduction in BMI. Educational level was categorized from self-reported highest education into “low” (primary school), “middle” (secondary/middle school or apprenticeship), and “high” (college or university). Pack-years of cigarettes smoked in life were computed from self-reported number of cigarettes smoked per day and smoking history. Physical activity was dichotomized from self-reported frequency and duration of moderate and vigorous physical activity into “sufficiently active” [either moderate physical activity ≥ 150 min/week, vigorous physical activity ≥ 60 min/week, or combined duration (duration of moderate physical activity + 2 × duration of vigorous physical activity) ≥ 150 min/week] and “insufficiently active” (otherwise).

In order to confirm that BMI is related to chronic inflammation, we examined the association between BMI and high-sensitive C-reactive protein (hs-CRP) within the study subjects (*n* = 206; one subject was excluded due to missing information on hs-CRP). We used information on both BMI and hs-CRP at SAPALDIA2 because hs-CRP was measured only at SAPALDIA2. Log-transformed hs-CRP was regressed on BMI after adjustment for age, sex, education level, study area, and pack-years of cigarettes smoked up to SAPALDIA2.

### 2.3. Methylome

Peripheral blood samples had been collected at SAPALDIA3, the second follow-up visit in 2010 of the cohort study. Pre-analytically, the blood samples were processed, and the buffy coat fraction was archived at −80 °C for five years until DNA extraction using the QIAamp Blood Mini Kit (QIAGEN, Hilden, Germany) following the manufacturer’s instructions [24]. A small number of samples yielded limited DNA quantity and were replaced by DNA extracted [using the Gentra Puregene Blood Kit (QIAGEN, Hilden, Germany) following the manufacturer’s instructions] from whole blood of the same venipuncture used for buffy coats. Bisulfite conversion of 600 ng of each sample was performed using the EZ-96 DNA Methylation-Gold™ Kit according to the manufacturer’s protocol (Zymo Research, Orange, CA, USA). Then, 200 ng of bisulfite-converted DNA was used for hybridization on the Illumina Infinium HumanMethylation450 BeadChip (Illumina, San Diego, CA, USA) following the Illumina Infinium HD Methylation protocol. Each array consisted of 96 samples distributed equally among 8 chips. The arrays were designed such that batch effects (e.g., sample position and intra- and inter-variability in arrays and chips) did not completely confound with biological covariates. This design allowed the retention of biological variation (including the variable of interest) after correction for technical variation. Specifically, each chip incorporated proportional amounts of samples representing the different centers, confounding factors, and cases-control status. Cases and controls were also placed on the chips (not following a specific sequence) in order to minimize technical variation between them. Raw fluorescence intensities were retrieved and preprocessed using the R package “*minfi*” [25]. One sample with sex mismatch was excluded. Background correction and dye bias correction were performed using Noob (normal-exponential out-of-band) procedure [26]. DNA methylation levels were expressed as β values, defined as the ratio of methylated intensity over total intensity with offset = 100. β values were set to missing if the detection *p*-value was higher than 10^−16^. Probes on sex chromosome were excluded. Probes were then filtered by call rate <0.95. All samples had call rate >0.95. Beta-mixture quantile normalization (BMIQ) procedure was conducted to correct for the Illumina probe design bias [27]. The probes known to hybridize with multiple genomic locations or to target CpG sites overlapping known SNPs with minor allele frequency greater than 1% in Europeans were excluded [28]. Finally, 430,591 CpGs were ready for analysis. In addition, principal component analysis (PCA) was conducted on the 220 control probes incorporated on the Illumina chip following Lehne and Drong [29], and β values were regressed on the first 30 components. All of the statistical analyses used the resulting residuals in place of the β values to account for batch effects.

### 2.4. EWIS of DNA Methylation and BMI on Adult-Onset Asthma

Logistic regression models were fitted for adult-onset asthma status on BMI at SAPALDIA3, residual of the β value at each CpG site, and their multiplicative interaction upon adjustment for age (in years), sex, education level, study area, pack-years of cigarettes smoked in life, bench time (in minutes), and Houseman estimates [30] of white blood cell composition for B cells, CD4 T cells, CD8 T cells, natural killer cells, monocytes, and eosinophils.

$$\begin{array}{c} Asthma\;\sim \;BMI\times Residual_{i}+Age+Sex+Education+Area+Packyear+Benchtime+Bcell\\ +CD4T+CD8T+NK+Mono+Eos\;\left(i=1,\;\ldots ,\;430591\right) \end{array}$$

We did not adjust for neutrophils because immune response in non-atopic participants was possibly driven by neutrophil proliferation [31,32], and therefore adjustment for neutrophils could obscure the association of interest. Despite the female preponderance in cases compared to controls (Table 1), we did not consider stratification or effect modification by sex based on our observation that the association between BMI and non-atopic adult-onset asthma did not differ by sex (Table S3). The interaction was considered genome-wide significant when the *p*-value from the interaction term was smaller than 0.1 after the Benjamini-Hochberg correction for multiple testing. As a sensitivity analysis, the same EWIS was repeated after further adjustment, either for physical activity or for neutrophil estimates.

### 2.5. EWIS of DNA Methylation and BMI Change on Adult-Onset Asthma

Logistic regression models were fitted for adult-onset asthma status on BMI change, residual of the β value at each CpG site, and their multiplicative interaction after adjustment for the same set of covariates as above and additionally for BMI at SAPALDIA2.

$$\begin{array}{c} Asthma\;\sim \;\left(BMI_{S3}-BMI_{S2}\right)\times Residual_{i}+BMI_{S2}+Age+Sex+Education+Area+Packyear\\ +Benchtime+Bcell+CD4T+CD8T+NK+Mono+Eos\;\left(i=1,\;\ldots ,\;430591\right) \end{array}$$

The same sensitivity analyses were conducted as above.

### 2.6. Candidate Pathway Enrichment Analyses Using Weighted Kolmogorov-Smirnov (WKS) Method

Genes relevant to the NLRP3-IL1B-IL17 axis were curated based on Kim et al. [13]. Inflammation-related genes were curated previously by Loza et al. into 17 mutually exclusive pathways [33]. The complete list of the genes assigned to each pathway can be found in Table S1. CpG sites were then assigned to the pathway if the CpG sites resided within 200 bp upstream or downstream of the genes included in each pathway.

We tested if the pathways were over-represented in the EWIS results by applying the Weighted Kolmogorov-Smirnov (WKS) enrichment test [34]. Using this algorithm, the absolute Z-statistics of the CpG sites assigned to each pathway (e.g., 219 CpG sites assigned to NLRP3-IL1B-IL17 axis) were compared with the null distribution created by 10,000 Monte-Carlo simulations of the absolute Z-statistics from the entire 430,591 CpG sites. In this approach, Z-statistics from all CpGs mapped to a pathway were compared to the null distribution without selection based on EWIS-derived *p*-values. Over-representation of the pathway was determined by Kolmogorov-Smirnov tests. Pathways with WKS *p*-value < 0.05 were declared as enriched. The procedure included permutation-based multiple testing correction [34,35].

### 2.7. Identification of Differentially Methylated Regions (DMR)

We used the R package DMRcate to identify DMRs [36]. The Z-statistics from EWIS were squared and smoothed using a Gaussian kernel with a bandwidth of 1000 bp and scaling factor of 2, which is equivalent to the kernel standard deviation of 500 bp. *p*-values were computed for each CpG site by comparison to the null distribution of the smoothed estimates. The regions containing at least one CpG site with Benjamini-Hochberg adjusted *p*-value < 0.05 were declared as significant. The significant DMRs were annotated to the genes whose promoter region, defined as 2000 bp from the transcription start site, overlapped with the DMRs.

### 2.8. Agnostic Pathway Enrichment Analyses Using Ingenuity Pathway Analysis (IPA)

The 1305 genes annotated to the 1131 DMRs identified as significant effect modification of BMI on adult-onset asthma were tested for over-representation using Ingenuity Pathway Analysis (IPA) (http://www.ingenuity.com/; QIAGEN, Redwood City, CA, USA) canonical pathway analysis. In brief, the maximum effect modification estimate and the minimum Benjamini-Hochberg adjusted *p*-value for each DMR were assumed to represent the expression level and the *p*-value, respectively, for the gene annotated to the DMR. The DMRs annotated multiple genes constituted multiple entries and each annotated a single gene. The DMRs with no gene annotation were excluded (*n* = 114). The 20 genes annotated to the 18 DMRs for BMI change were too few to conduct the same pathway analysis.

## 3. Results

**Figure 1:**
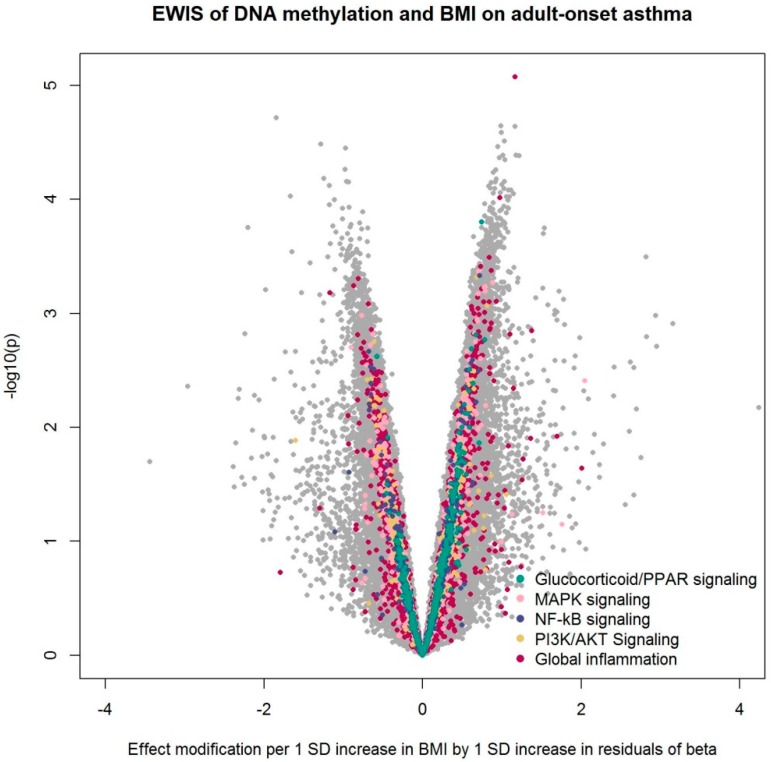
Volcano plot from the epigenome-wide interaction study (EWIS) of DNA methylation and BMI on adult-onset asthma. The EWIS fitted logistic regression models of adult-onset asthma on BMI, residuals of DNA methylation at a single CpG site, and their multiplicative interaction upon adjustment for age, sex, education level, study area, pack-years of cigarettes smoked in life, bench time, and white blood cell composition estimates for B cells, CD4 T cells, CD8 T cells, natural killer cells, monocytes, and eosinophils. The CpGs assigned to the pathway enriched with *p* < 0.05 are highlighted in colors. No line of significance was drawn, as no CpG reached genome-wide significance after multiple testing corrections.

**Figure 2:**
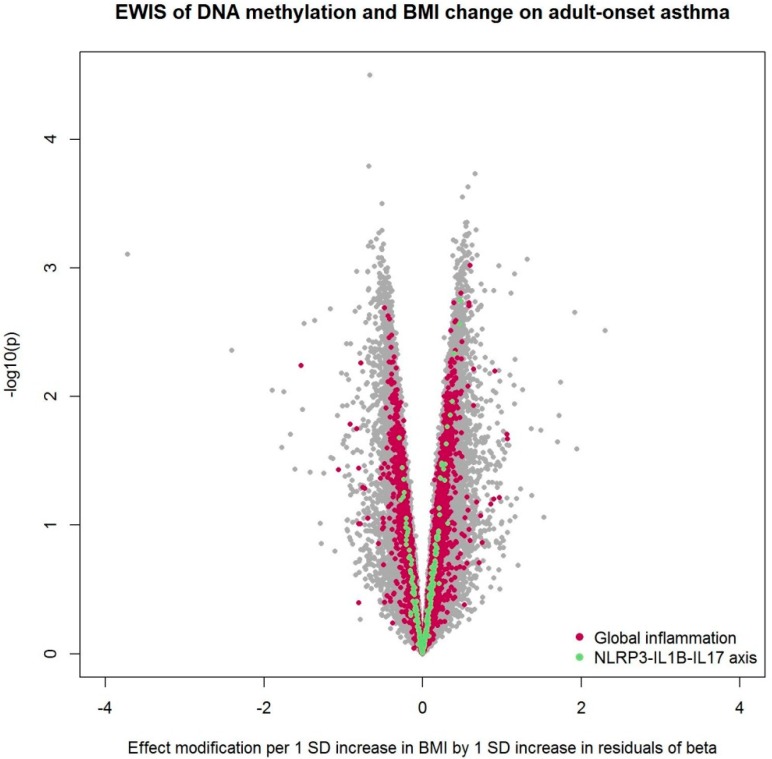
Volcano plot from the EWIS of DNA methylation and BMI change on adult-onset asthma. The EWIS fitted logistic regression models of adult-onset asthma on BMI change, residuals of DNA methylation at a single CpG site, and their multiplicative interaction upon adjustment for age, sex, education level, study area, pack-years of cigarettes smoked in life, bench time, and white blood cell composition estimates for B cells, CD4 T cells, CD8 T cells, natural killer cells, monocytes, and eosinophils. The CpGs assigned to the pathway enriched with *p* < 0.05 are highlighted in colors. No line of significance was drawn, as no CpG reached genome-wide significance after multiple testing corrections.

From association analysis using SAPALDIA2 information, we confirmed a positive association between BMI and hs-CRP. One unit increase in BMI was associated with 0.1 unit increase in log-transformed hs-CRP (95% confidence interval [0.07, 0.14]; *p* < 10^−8^). We conducted an EWIS of DNA methylation and BMI or BMI change over 10 years on adult-onset asthma among non-atopic, non-smoking SAPALDIA3 participants (Table 1). We found no epigenome-wide significant effect modification after multiple testing corrections. Sensitivity analyses with additional adjustment for physical activity or neutrophil estimates also resulted in no epigenome-wide significant CpG sites. Figure 1, Figure 2, and Figures S1–S4 summarize the EWIS results.

After pathway enrichment analysis of 17 a priori curated inflammatory pathways [33], we found an over-representation of effect modification by DNA methylation of BMI on adult-onset asthma in several pathways: Glucocorticoid/PPAR (peroxisome proliferator-activated receptor) signaling, MAPK (mitogen-activated protein kinase) signaling, NF-κB (nuclear factor kappa-B) signaling, and PI3K/AKT (phosphatidylinositol-3-kinases/protein kinase B) signaling (Table 2). The pathway “global inflammation”, defined as the entirety of the 1027 genes assigned to the 17 inflammation pathways, also showed enrichment. In the sensitivity analyses, the enrichment of PI3K/AKT signaling disappeared after adjustment for physical activity, while the enrichment of NF-κB signaling and PI3K/AKT signaling disappeared after adjustment for neutrophil estimates (Table 2).

When the EWIS was conducted using BMI change instead of BMI, NLRP3-IL1B-IL17 axis and global inflammation were enriched (Table 3). No enrichment was found after additional adjustment for physical activity. Global inflammation remained enriched after adjustment for neutrophil estimates, while the NLRP3-IL1B-IL17 axis did not. Table 2 and Table 3 summarize the WKS enrichment test results.

**Figure 3:**
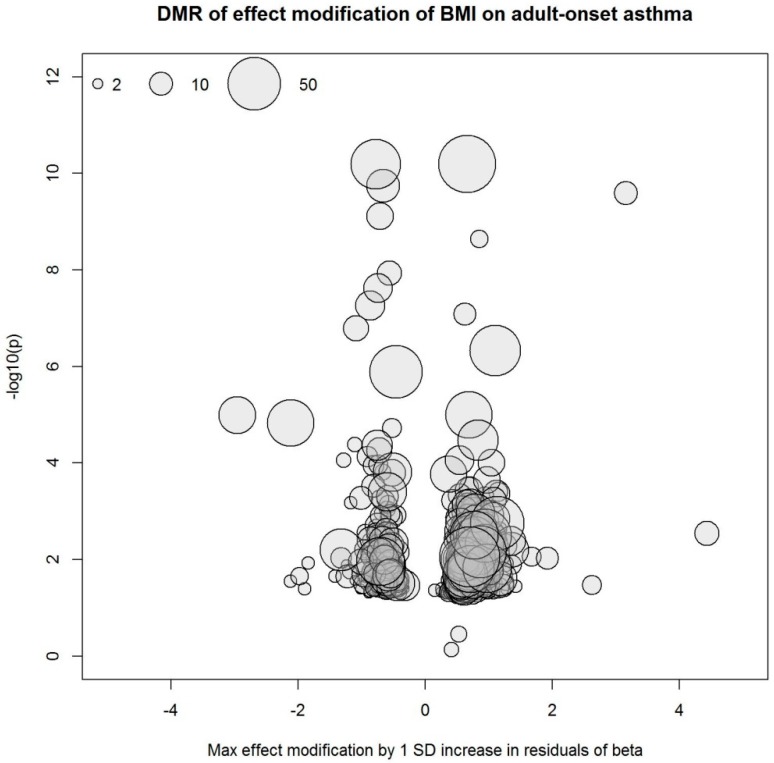
Differentially methylated regions (DMRs) derived from the EWIS of DNA methylation and BMI on adult-onset asthma. Circle size represents the number of CpG sites in the region.

**Figure 4:**
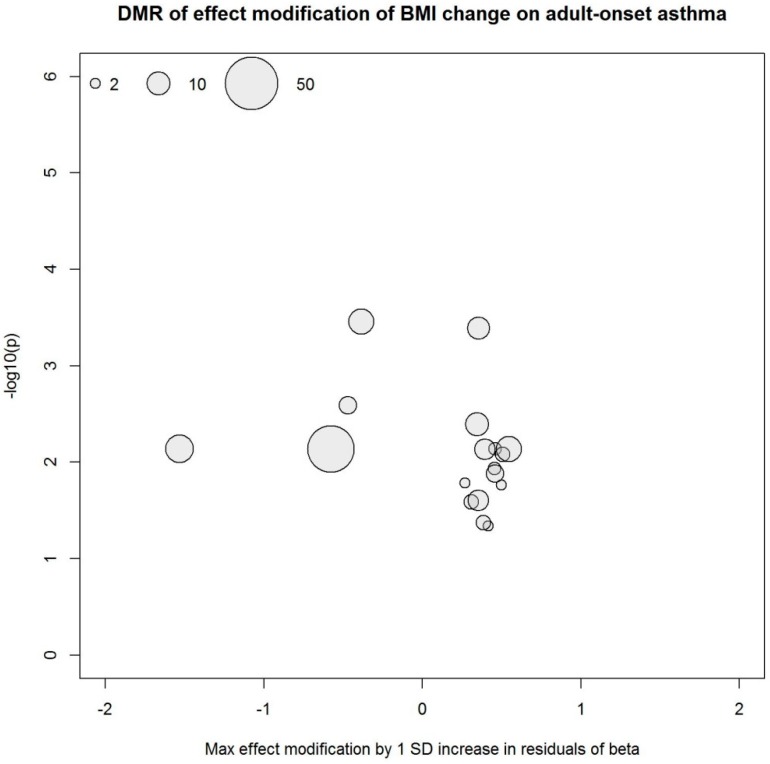
DMRs derived from the EWIS of DNA methylation and BMI change on adult-onset asthma. Circle size represents the number of CpG sites in the region.

Our study was likely underpowered to identify differential methylation markers from the EWIS approach considering the large dimension of the methylome data, the relatively small sample size, and the investigation of effect modification instead of main effects. Acknowledging these issues, we additionally searched for differentially methylated regions (DMR) using the R package DMRcate [36]. Based on the EWIS, we identified 1131 DMRs that modify the association of BMI with non-atopic asthma as well as 18 DMRs that interact with BMI change, affecting its association with non-atopic asthma. Figure 3 and Figure 4 summarize the DMRs. Each circle represents one DMR whose x- and y-coordinates depict maximum effect modification by 1 SD increase in residuals within the region and minimum Benjamini-Hochberg adjusted *p*-value within the region, respectively. The 1131 and 18 DMRs were annotated to 1305 and 20 genes, respectively, and there were two overlapping genes. In an agnostic pathway enrichment analysis, using IPA, we found PPARα/RXRα (retinoid X receptor alpha) activation (*p* = 0.015), ERK (extracellular-regulated kinase) /MAPK signaling (*p* = 0.038), and glucocorticoid receptor signaling (*p* = 0.038), among others, enriched for the 1305 genes annotated to the 1131 DMRs. Figure S5 and Table S2 summarize the IPA pathway analysis results.

## 4. Discussion

We found no single CpG sites of genome-wide significant effect modification. However, we did find DMRs and pathway enrichments. DNA methylation markers usually act in concert at neighboring CpG sites [37,38], therefore EWIS alone may fail to identify true differential methylation markers [39].

Global inflammation, defined as the entirety of the 1027 inflammation-related genes according to the classification proposed by Loza et al. [33], was over-represented in the DNA methylation signals modifying the BMI-adult-onset asthma association. The agnostic search for the pathway enrichment of the DMRs also revealed several relevant pathways. Our study results are consistent with inflammation modifying the effect of BMI on adult-onset non-atopic asthma. Adiposity is believed to induce chronic systemic inflammation via dysregulated production of pro-inflammatory cytokines and immune cells infiltrated into adipose tissue [11,40]. Our findings suggest that altered methylation in pro-inflammatory gene networks potentially mediate the link between overweight and non-atopic adult-onset asthma. This is in line with previous findings in children. Rastogi and her colleagues reported hypomethylation in the promoter of genes involved in innate immunity and non-atopic inflammation in obese children with asthma [20].

Among the 17 inflammation pathways curated by Loza and his colleagues [33], glucocorticoid/PPAR signaling showed the strongest enrichment. The agnostic search for the pathway enrichment in the DMRs also found enrichment of Glucocorticoid receptor signaling in addition to PPARα/RXRα activation. The glucocorticoid/PPAR signaling includes the genes coding for the nuclear receptors for glucocorticoids, PPARs, and associated proteins. PPARs have been associated with asthma, and PPAR agonists are being considered as a new asthma treatment [41]. While we cannot rule out the possibility that asthma medication led to DNA methylation on the genes involved in glucocorticoid receptor signaling, it is not likely that this would explain the interaction of methylation signals in this pathway with BMI or BMI change.

MAPK signaling, NF-κB signaling, and PI3K/AKT signaling are all involved in signal transduction downstream to the detection of insults, e.g., by TLR (toll-like receptor). The enrichment signals for PI3K/AKT signaling disappeared when the model was additionally adjusted for physical activity, suggesting that the effect modification of BMI on adult-onset asthma might be confounded or mediated by physical activity. The enrichment of NF-κB signaling and PI3K/AKT signaling disappeared when the model was further adjusted for neutrophil estimates, suggesting that the effect modification in this pathway might be modulated by neutrophil proliferation and hence disguised by the adjustment for neutrophil estimates. ERK/MAPK signaling was also over-represented in the DMRs from the agnostic pathway analysis.

Interestingly, NLRP3-IL1B-IL17 axis was enriched in the EWIS using BMI change, suggesting that BMI change represents a phenotype distinct from BMI. The well-known limitation of BMI is that it cannot distinguish fat from lean mass. Weight change in late adulthood is more likely attributable to change in fat than in lean mass [42], and people tend to lose lean mass while aging [43,44]. Therefore, fat composition could be better reflected in BMI change than in BMI. Our finding that the enrichment of NLRP3-IL1B-IL17 axis disappeared upon adjustment for neutrophil estimates is consistent with the growing evidence of IL17 playing a role in recruitment, accumulation, and survival of neutrophils in asthma [31,32].

The NLRP3 inflammasome and downstream activity have been associated with both asthma [45] and obesity [46] in humans. To the best of our knowledge, however, this study is the first to provide evidence of the NLRP3-IL1B-IL17 axis as a link between overweight and non-atopic adult-onset asthma in humans. This study is also the first to provide evidence that inflammation represented in the DNA methylation profile may play a role in the link between overweight and non-atopic adult-onset asthma.

Pathway enrichment analyses have often been applied to interpret genome-wide patterns of differential methylation. Widely used tools for pathway enrichment analyses include GSEA (gene set enrichment analysis) [47], DAVID (the database for annotation, visualization, and integrated discovery) [48], and IPA (http://www.ingenuity.com; QIAGEN, Redwood City, CA, USA), which were originally developed to analyze differential expression of genes. In order to apply these tools to epigenetics, differential methylation signals first need to be translated from CpGs to genes. This can lead to a bias, e.g., that large genes with multiple CpGs are more likely to be represented. In this study, we applied the WKS method to test pathway enrichment of the EWIS results. The WKS method works in a similar way as GSEA, but the enrichment is quantified using CpG (and not gene) level statistics. This method also supports examination of custom-curated pathways, allowing straightforward interpretation.

We were underpowered to identify differential DNA methylation as effect modifiers, although the problem was partly overcome by applying integrative approaches, i.e., DMR and pathway enrichment analysis. The fact that DMR analysis resulted in more than 1000 signals while EWIS identified no signals indicates that a multivariate approach is better suited than an univariate approach to study epigenetic marks that function in clusters. We applied the WKS enrichment analyses to the absolute Z-statistics. Therefore, the direction of the effect modification, meaning whether hypo- or hyper-methylation was associated with increased effect of BMI, was not taken into consideration. The IPA pathway analysis results may have been biased by transforming the DMRs into gene-level statistics. Moreover, we annotated CpG sites simply based on the location regardless of their functional information, i.e., whether they resided in promoter, gene body, or intergenic region, CpG islands or not, etc. The cross-sectional design of our study along with the effect modification being studied without mediation analysis prevents inference of causal mediation. However, our findings of the enriched pathways using BMI change cannot be driven entirely by reverse causality because BMI change preceded the DNA methylation measurements. Given the recent Mendelian randomization studies that reported a causal effect of BMI on childhood asthma [49] and a causal effect of BMI on lifetime asthma [50], the overweight-asthma association may potentially be causal. In this study, we observed differential DNA methylation enriched in inflammatory pathways but did not measure chronic inflammation directly in the study subjects. However, we confirmed that the study subjects showed a strong positive association between BMI and hs-CRP at SAPALDIA2. In order to elucidate if overweight-induced inflammation causes asthma, further studies, including two-step Mendelian randomization studies, are warranted. Taking transcriptomics and proteomics study of blood, lung, and adipose tissue with asthma phenotype heterogeneity into consideration will be crucial.

## 5. Conclusions

DNA methylation measured in peripheral blood is consistent with inflammation as a potential link between BMI and adult-onset asthma and with the NLRP3-IL1B-IL17 axis as a potential link between BMI change over 10 years and adult-onset asthma in non-atopic non-smokers.

## Figures and Tables

**Table 1:** Study samples’ characteristics by adult-onset asthma status at the Swiss Cohort Study on Air Pollution and Lung and Heart Diseases in Adults 2010–11 (SAPALDIA3).

	Cases	Controls
**N**	61	146
Age (year)	60.8 (15.6)	57.4 (15.0)
Female	43 (70%)	82 (56%)
BMI (kg/m^2^) ^a^	25.7 (5.8)	24.5 (4.8)
BMI change (kg/m^2^) ^b^	0.4 (2.0)	0.5 (1.6)
Smoking ^c^		
Former	27 (44%)	50 (34%)
Never	34 (56%)	96 (66%)
Pack-years ^d^	7.8 (13.3)	6.8 (11.6)
Education level ^e^		
Low	0 (0%)	2 (1%)
Middle	43 (70%)	94 (64%)
High	18 (30%)	50 (34%)
Physical activity ^f^		
Insufficiently active	18 (30%)	30 (21%)
Sufficiently active	42 (69%)	113 (77%)
N/A	1 (2%)	3 (2%)
Bench time (min) ^g^	80.0 (34.0)	82.5 (32.5)
hs-CRP (mg/L) ^h^	1.3 (1.4)	0.7 (1.2)

Data are presented as count (%) or median (interquartile range). ^a^ Measured at SAPALDIA3. ^b^ Change in body mass index (BMI) between SAPALDIA 2001–3 (SAPALDIA2) and SAPALDIA3. ^c^ Former smokers had not smoked for at least 10 years before blood was drawn. ^d^ Only computed in former smokers (pack-years were set to zero for never smokers). ^e^ Low = primary school; middle = secondary/middle school or apprenticeship; high = college or university. ^f^ Sufficiently active at SAPALDIA3 = either moderate physical activity ≥ 150 min/week, vigorous physical activity ≥ 60 min/week, or combined duration (duration of moderate physical activity + 2 × duration of vigorous physical activity) ≥ 150 min/week; insufficiently active = otherwise. N/A = not available. ^g^ Time elapsed between blood draw and storage in freezer. ^h^ Measured at SAPALDIA2.

**Table 2:** EWIS of DNA methylation and BMI on adult-onset asthma. Enrichment test results for 17 inflammation pathways and NLRP3-IL1B-IL17 axis.

Pathway	#Genes	#CpGs	Enrichment *p*-Value		
			Basic Model	Adjusted for Physical Activity	Adjusted for Neutrophil Counts
Adhesion-extravasation-migration	142	1737	0.48	0.30	0.37
Apoptosis signaling	68	1210	0.22	0.34	0.32
Calcium signaling	14	413	0.81	0.72	0.70
Complement cascade	40	483	0.92	0.73	0.96
Cytokine signaling	172	1883	0.070	0.053	0.067
Eicosanoid signaling	39	450	0.58	0.78	0.55
Glucocorticoid/PPAR signaling	21	404	**0.0023**	**0.0053**	**0.0039**
G-Protein coupled receptor signaling	42	1133	0.74	0.49	0.66
Innate pathogen detection	50	515	0.89	0.72	0.88
Leukocyte signaling	121	1429	0.14	0.059	0.090
MAPK signaling	118	2682	**0.013**	**0.0036**	**0.018**
Natural killer cell signaling	31	368	0.54	0.41	0.51
NF-κB signaling	33	654	**0.031**	**0.0028**	0.054
Phagocytosis-Ag presentation	39	1058	0.81	0.72	0.66
PI3K/AKT signaling	37	907	**0.031**	0.23	0.053
ROS/glutathione/cytotoxic granules	22	190	0.58	0.45	0.53
TNF superfamily signaling	38	537	0.78	0.69	0.73
Global inflammation ^§^	1027	15985	**0.0026**	**0.011**	**0.0057**
NLRP3-IL1B-IL17 axis	11	219	1.00	0.99	1.00

The basic model regressed adult-onset asthma on BMI, residuals of DNA methylation at a single CpG site, and their multiplicative interaction upon adjustment for age, sex, education level, study area, pack-years of cigarettes smoked in life, bench time, and white blood cell composition estimates for B cells, CD4 T cells, CD8 T cells, natural killer cells, monocytes, and eosinophils. ^§^ Total of the 17 inflammation pathways; the number of CpG in this pathway (15,985) is smaller than the sum of the CpGs assigned to 17 pathways because there are CpGs assigned to multiple pathways, although the 17 pathways are mutually exclusive at gene level. Enrichment *p*-values are in bold if *p* < 0.05.

**Table 3:** EWIS of DNA methylation and BMI change on adult-onset asthma: enrichment test results for 17 inflammation pathways and NLRP3-IL1B-IL17 axis.

Pathway	#Genes	#CpGs	Enrichment *p*-Value		
			Basic Model	Adjusted for Physical Activity	Unadjusted for Neutrophil Counts
Adhesion-extravasation-migration	142	1737	0.67	0.60	0.39
Apoptosis signaling	68	1210	0.50	0.37	0.22
Calcium signaling	14	413	0.29	0.34	0.21
Complement cascade	40	483	0.45	0.64	0.34
Cytokine signaling	172	1883	0.26	0.35	0.21
Eicosanoid signaling	39	450	0.48	0.17	0.61
Glucocorticoid/PPAR signaling	21	404	0.063	0.15	0.072
G-Protein coupled receptor signaling	42	1133	0.47	0.88	0.46
Innate pathogen detection	50	515	0.059	0.12	0.13
Leukocyte signaling	121	1429	0.35	0.49	0.34
MAPK signaling	118	2682	0.13	0.33	0.24
Natural killer cell signaling	31	368	0.91	0.75	0.91
NF-κB signaling	33	654	0.70	0.49	0.62
Phagocytosis-Ag presentation	39	1058	0.51	0.89	0.71
PI3K/AKT signaling	37	907	0.98	0.98	0.89
ROS/glutathione/cytotoxic granules	22	190	0.24	0.55	0.14
TNF superfamily signaling	38	537	0.085	0.33	0.065
Global inflammation ^§^	1027	15985	**0.048**	0.23	**0.028**
NLRP3-IL1B-IL17 axis	11	219	**0.046**	0.13	0.15

The basic model regressed adult-onset asthma on BMI change, residuals of DNA methylation at a single CpG site, and their multiplicative interaction upon adjustment for BMI at SAPALDIA2, age, sex, education level, study area, pack-years of cigarettes smoked in life, bench time, and white blood cell composition estimates for B cells, CD4 T cells, CD8 T cells, natural killer cells, monocytes, and eosinophils. ^§^ Total of the 17 inflammation pathways; the number of CpG in this pathway (15,985) is smaller than the sum of the CpGs assigned to 17 pathways because there are CpGs assigned to multiple pathways, although the 17 pathways are mutually exclusive at gene level. Enrichment *p*-values are in bold if *p* < 0.05.

## Supplementary Materials

The following are available online at http://www.mdpi.com/1660-4601/16/4/600/s1, Table S1: Genes curated to 17 inflammatory pathways and NLRP3-IL1B-IL17 axis, Table S2: Agnostic pathway enrichment results of the DMRs identified from the EWIS of DNA methylation and BMI on adult-onset asthma, Table S3: Sex difference in BMI effect on adult-onset asthma, Figure S1: Volcano plot from the EWIS of DNA methylation and BMI on adult-onset asthma, further adjusted for physical activity, Figure S2: Volcano plot from the EWIS of DNA methylation and BMI on adult-onset asthma, further adjusted for neutrophil estimates, Figure S3: Volcano plot from the EWIS of DNA methylation and BMI change on adult-onset asthma, further adjusted for physical activity, Figure S4: Volcano plot from the EWIS of DNA methylation and BMI change on adult-onset asthma, further adjusted for neutrophil estimates, Figure S5: Agnostic pathway enrichment results of the DMRs identified from the EWIS of DNA methylation and BMI on adult-onset asthma.
