# Supplementary material for: DNA Methylation in Inflammatory Pathways Modifies the Association between BMI and Adult-Onset Non-Atopic Asthma
Source: Int J Environ Res Public Health. 2019 Feb 19;16(4):600. doi: 10.3390/ijerph16040600 (PMC6406386; doi:10.3390/ijerph16040600)
Supplement: Supplementary file 1 [file ijerph-16-00600-s001.pdf]

Supplementary Material

DNA Methylation in Inflammatory Pathways Modifies the Association between BMI and Adult-Onset Non-Atopic Asthma

Ayoung Jeong [1,2], Medea Imboden [1,2], Akram Ghantous [3], Alexei Novoloaca [3], Anne-Elie Carsin [4,5,6], Manolis Kogevinas [4,5,6], Christian Schindler [1,2], Gianfranco Lovison [7], Zdenko Herceg [3], Cyrille Cuenin [3], Roel Vermeulen [8], Deborah Jarvis [9], André F. S. Amaral [9], Florian Kronenberg [10], Paolo Vineis [11,12] and Nicole Probst-Hensch [1,2,*]

1 Swiss Tropical and Public Health Institute, 4051 Basel, Switzerland; a.jeong@swisstph.ch (A.J.); medea.imboden@swisstph.ch (M.I.); christian.schindler@swisstph.ch (C.S.)
2 Department of Public Health, University of Basel, 4001 Basel, Switzerland
3 International Agency for Research on Cancer, 69372 Lyon, France; ghantousa@iarc.fr (A.G.); NovoloacaA@students.iarc.fr (A.N.); hercegz@iarc.fr (Z.H.); CueninC@iarc.fr (C.C.)
4 ISGlobal, Barcelona Institute for Global Health, 08003 Barcelona, Spain; anneelie.carsin@isglobal.org (A.-E.C.); manolis.kogevinas@isglobal.org (M.K.)
5 Universitat Pompeu Fabra (UPF), 08002 Barcelona, Spain
6 CIBER Epidemiología y Salud Pública (CIBERESP), 08005 Barcelona, Spain
7 Department of Economics, Business and Statistics, University of Palermo, 90128 Palermo, Italy; gianfranco.lovison@unipa.it
8 Environmental Epidemiology Division, Utrecht University, Institute for Risk Assessment Sciences, 3584CM Utrecht, Netherlands; R.C.H.Vermeulen@uu.nl
9 Population Health and Occupational Disease, National Heart and Lung Institute, Imperial College, SW3 6LR London, UK; d.jarvis@imperial.ac.uk (D.J.); a.amaral@imperial.ac.uk (A.F.S.A.)
10 Division of Genetic Epidemiology, Medical University of Innsbruck, 6020 Innsbruck, Austria; Florian.Kronenberg@i-med.ac.at
11 MRC-PHE Centre for Environment and Health, School of Public Health, Imperial College London, W2 1PG London, UK; p.vineis@imperial.ac.uk
12 Italian Institute for Genomic Medicine (IIGM), 10126 Turin, Italy
* Correspondence: nicole.probst@swisstph.ch; Tel.: +41-61-284-8378

Table S1. Genes curated to 17 inflammatory pathways and NLRP3-IL1B-IL17 axis.

Pathway	#Genes	Genes
Adhesion-extravasation-migration	142	PTPRU, VCAM1, CD58, CD2, MUC1, DARC, CD48, F11R, XCL1, SELP, SELL, SELE, RASSF5, CD34, MENA, ITGB1, CXCL12, VCL, ADAM8, ARHGAP1, CTNND1, SIPA1, CTTN, FUT4, MMP7, MMP10, MMP1, MMP12, BLR1, THY1, JAM3, CD9, ITGB7, ITGA5, MMP19, MYL6, SELPLG, PXN, MMP14, SPN, ITGAL, ITGAM, ITGAX, ITGAD, MLCK, MMP2, CCL22, CX3CL1, CCL17, CDH5, CKLF, CRK, ITGAE, CXCL16, MYH10, CCL2, CCL7, CCL11, CCL8, CCL13, CCL1, CCL5, CCL16, CCL15, CCL23, CCL18, CCL4, CCR7, ITGA2B, ITGB3, ITGA3, ICAM2, PECAM1, ITGB4, CD226, ICAM1, ICAM5, ICAM3, CD97, CEACAM5, CEACAM6, CEACAM3, CEACAM1, CEACAM8, PLAUR, VASP, CD33, ROCK2, ITGA6, ITGA4, ITGAV, ALS2, CCL20, SIGLEC1, MMP9, JAM2, ITGB2, MIF, MYH9, CCR4, CX3CR1, CCR8, CCBP2, CXCR6, XCR1, CCR1, CCR3, CCR2, CCR5, CCRL2, RHOA, ALCAM, CD47, CD96, RHOH, CXCL1, PPBP, CXCL5, CXCL3, CXCL2, CXCL9, CXCL10, CXCL11, CXCL13, FYB, CCL28, ITGA1, ITGA2, CXCL14, LECT2, HMMR, NT5E, VIL2, CCR6, CCL26, CCL24, PTP-PEST, CD36, CCL27, CCL19, CCL21, CD99
Apoptosis signaling	68	DFFB, DFFA, CASP9, MCL1, DAP3, LMNA, FASLG, CAPN2, PARP1, CDC2, FAS, CASP7, GAS2, BAD, CAPN1, FADD, BIRC3, BIRC2, CASP12, BCL2L14, CRADD, APAF1, DIABLO, ACIN1, BCL2L2, RIPK3, BCL2L10, BCL2A1, TP53, ROCK1, BCL2, CASP14, CAPNS1, BBC3, BAX, BCL2L12, HTRA2, BCL2L11, TANK, CFLAR, CASP10, CASP8, CAPN10, BCL2L1, BCL2L13, BID, A4GALT, BIK, FAIM, TNFSF10, PTPN13, CASP6, CASP3, DAP, DAXX, BAK1, TNFRSF21, CASP8AP2, CYCS, CASP2, TNFRSF10B, TNFRSF10C, TNFRSF10D, TNFRSF10A, RIPK2, DAPK1, SPTAN1, ENDOG
Calcium signaling	14	CAMK1D, PPP3CB, NFATC4, AKAP5, NFATC3, NFAT5, NFATC1, PPP3R1, NFATC2, CABIN1, PPP3CA, CAMK4, PPP3CC, PPP3R2
Complement cascade	40	MASP2, C1QA, C1QC, C1QB, C8A, C8B, SERPINC1, CFH, C4BPB, C4BPA, CD55, CR2, CR1, CD46, MBL2, CD59, SERPING1, C1S, C1R, C3AR1, SERPINA1, SERPINA5, SERPINF2, CFD, C3, CPAMD8, C5AR1, SERPIND1, MASP1, CFI, C9, C7, C6, C2, CFB, C4B, SERPINE1, C5, C8G, PFC
Cytokine signaling	172	IL22RA1, TXLNA, CSF3R, JAK1, IL23R, IL12RB2, CSF1, PIAS3, S100A9, S100A12, S100A8, ILF2, IL6R, CRP, IL10, IL19, IL20, IL24, IRF6, TGFB2, IL15RA, IL2RA, GATA3, BMPR1A, IRF7, CASP1, IL18, IL10RA, STAT2, STAT6, IFNG, IL26, IL22, SOCS2, IL31, IL17D, HMGB1, IL25, ISGF3G, BMP4, SOCS4, TGFB3, PIAS1, IL16, IL32, SOCS1, IL4R, IL21R, IRF8, IL17C, CSF3, STAT5B, STAT5A, STAT3, TBX21, ACE, SOCS3, TGIF, PTPN2, PIAS2, SOCS6, PIAS4, EBI3, C19ORF10, TYK2, IL27RA, JAK3, IL12RB1, IL28B, IL28A, IL29, TGFB1, IRF3, IL11, ADAM17, SOCS5, TGFA, IL1R2, IL1R1, IL1RL2, IL1RL1, IL18R1, IL18RAP, IL1A, IL1B, IL1F7, IL1F9, IL1F6, IL1F8, IL1F5, IL1F10, IL1RN, NMI, STAT1, STAT4, BMPR2, IL8RB, IL8RA, BMP2, TGIF2, CEBPB, PTPN1, IFNAR2, IL10RB, IFNAR1, IFNGR2, IL17RA, LIF, OSM, CSF2RB, IL2RB, PDGFB, IL5RA, TGFBR2, CISH, IL17RB, IL12A, THPO, IL1RAP, PDGFRA, IL8, BMPR1B, IL2, IL21, IL15, IRF2, IL7R, LIFR, OSMR, IL31RA, IL6ST, IL3, CSF2, IRF1, IL5, IL13, IL4, IL9, IL17B, CSF1R, PDGFRB, IL12B, IRF4, AGER, VEGF, IL17A, IL17F, IFNGR1, IL6, IRF5, IL7, JAK2, C9ORF26, IFNB1, IFNW1, IFNA21, IFNA4, IFNA10, IFNA5, IFNA6, IFNA2, IFNA8, IFNA1, IFNK, NFIL3, TGFBR1, ENG, IL3RA, CRSP2, IL2RG, IL13RA2, IL13RA1

Table S1 (cont.) Genes curated to 17 inflammatory pathways and NLRP3-IL1B-IL17 axis.

Pathway	#Genes	Genes
Eicosanoid signalin g	39	PLA2G2A, PLA2G2D, PTGER3, PTGFR, MGST3, PTGS2, AKR1C3, ALOX5, GPR44, PTGES3, LTA4H, ALOX5AP, CYSLTR2, PTGDR, PTGER2, DPEP3, DPEP2, DPEP1, ALOX15, ALOX12, ALOX15B, ALOX12B, TBXA2R, PTGER1, PTGIR, FPRL1, FPRL2, PTGIS, GGT1, PGDS, MGST2, PTGER4, LTC4S, TBXAS1, PTGS1, PTGES2, PTGES, PTGDS, CYSLTR1
Glucocorticoid/ PPAR signaling	21	GMEB1, FAF1, FKBP4, NR4A1, PPARBP, NCOA1, NR4A2, SDPR, HSPD1, GMEB2, NRIP1, PPARA, KPNA1, PPARGC1A , NR2F1, NR3C1, FOXO3A, SGK, CITED2, GLCCI1, NCOA2
G-Protein coupled r eceptor signaling	42	PDE4B, PRKACB, ADORA3, RGS1, ADORA1, CREM, ADRA2A, PDE3B, PLCB3, ADRBK1, PDE2A, PDE3A, ADCY6, PDE1B, ADCY4, PLCB2, CREBBP, ADORA2B, PRKAR1A, PDE4A, PRKACA, PDE4C, PDE1A, CREB1, PLCB1, PLCB4, ADORA2A, HRH1, PRKAR2A, ADCY5, GRK4, ADRA2C, ADCY2, PDE4D, ADRB2, HRH2, SYNGAP1, PDE1C, ADCY1, PRKAR2B, PTK2B, PRKACG
Innate pathogen de tection	50	PGLYRP3, PGLYRP4, CIAS1, NALP6, TOLLIP, CASP5, COP1, ICEBERG, TIRAP, IRAK4, IRAK3, HSP90B1, OAS1, OAS2, PYCARD, CARD15, NALP1, SARM1, TICAM1, SITPEC, PGLYRP2, PGLYRP1, CARD8, NALP12, NALP2, CARD12, IFIH1, PRKRA, KIAA1271, LBP, IRAK2, MYD88, TLR9, TLR10, TLR1, TLR6, TLR2, TLR3, TLR5, CARD6, CD180, TICAM2, CD14, CARD4, LY96, DDX58, TLR4, TLR7, TLR8, IRAK1
Leukocyte signalin g	121	CD52, PTAFR, LCK, CD53, PTPN22, IGSF3, IGSF2, VTCN1, FCGR1C, FCER1A, SLAMF9, SLAMF6, SLAMF1, SLAMF7, FCER1G, FCGR2A, FCGR2B, SH2D1B, CD3Z, PTPRC, PTPN7, PIGR, TCF8, BLNK, PIK3AP1, CD44, RAG1, PTPRJ, MS4A2, MS4A1, CD5, SCGB1A1, SLC3A2, TCIRG1, CD3E, CBL, CD4, LRRC23, KITLG, FLT3, EDNRB, IGHA1, CSK, LAT, SLC7A5, SCARF1, CD68, CD79B, GRB2, SECTM1, VAV1, FCER2, CD22, CD79A, CD37, FLT3LG, SIGLEC10, SIGLEC5, LILRB3, LILRA6, LILRB2, LILRA3, LILRA5, LILRA4, LILRA2, LILRA1, LILRB4, LILRP2, FCAR, TACR1, CD8A, CD8B1, MAL, ZAP70, MARCO, DPP4, CD28, CTLA4, ICOS, SIRPB1, SIRPG, PTPNS1, CST7, SLA2, CD40, ICOSLG, SCARF2, VPREB1, IGLL1, GRAP2, MST1R, CD80, CD86, SH3BP2, CD38, TXK, TEC, DAPP1, EDNRA, TCF7, ITK, LCP2, SCGB3A1, TREM2, TREM1, TFEB, FYN, TRGV9, PILRB, PBEF1, MSR1, PAG1, PDCD1LG2, PAX5, SHB, SEMA4D, SYK, ABL1, FOXP3, BTK, CD40LG
MAPK signaling	118	PRKCZ, RAP1GAP, RPS6KA1, MAP3K6, HDAC1, MKNK1, JUN, RAP1A, NRAS, SHC1, MEF2D, IFI16, PLA2G4A, MAPKAPK2, ATF3, DUSP10, PRKCQ, MAPK8, HRAS, INS, MADD, RPS6KA4, FOSL1, PPP1CA, PAK1, PPP2R1B, HSPB2, ETS1, KRAS, RAPGEF3, ATF1, DDIT3, DUSP6, PPP1CC, MAPKAPK5, SOS2, PPM1A, MAX, FOS, RPS6KA5, RASGRP1, TLN2, MAP2K1, MEF2A, EEF2K, PRKCB1, MAPK3, BCAR1, MAP2K4, MAP2K3, KSR1, PRKCA, MAP2K6, MAP2K2, JUND, MEF2B, MAP4K1, PPP2R1A, YWHAQ, MYCN, PPP1CB, SOS1, PRKCE, DUSP2, ATF2, PPP1R7, SRC, PLCG1, YWHAB, ETS2, HMGN1, MAPK1, YWHAH, RAC2, MAP3K7IP1, ATF4, EP300, MAPK12, MAPK11, PPARG, RAF1, KCNH8, MAPKAPK3, PRKCD, PPP2R3A, EGF, MAP3K1, RASA1, MEF2C, HINT1, PPP2CA, PPP2R2B, DUSP1, MAPK9, MAPK14, MAPK13, MAP3K7, HDAC2, PTPRK, MAP3K5, MAP3K7IP2, ESR1, RAC1, EGFR, HSPB1, YWHAG, BRAF, DUSP4, PPP2CB, LYN, YWHAZ, MYC, PTK2, TLN1, RAPGEF1, ARAF, ELK1, DUSP9

Table S1 (cont.) Genes curated to 17 inflammatory pathways and NLRP3-IL1B-IL17 axis.

Pathway	#Genes	Genes
Natural killer cell si gnaling	31	CD160, CD244, FCGR3A, NCAM1, B3GAT1, PTPN6, KLRB1, KLRD1, KLRK1, KLRC4, KLRC2, KLRC1, PTPN11, CD300A, TYROBP, SIGLEC7, LAIR1, LILRB1, KIR3DL3, KIR2DS4, KIR3DL2, NCR1, HLA-G, HLA-E, MICA, MICB, NCR3, NCR2, RAET1E , ULBP3, SH2D1A
NF-κB signaling	33	BCL10, CHUK, BTRC, NFKB2, RELA, NFRKB, TBK1, UBE2N, NFKBIA, MEFV, CSNK2A2, MAP3K14, MAP3K3, CARD14, MALT1, MAP2K7, NFKBIB, BCL3, RELB, EIF2AK2, REL, CSNK2A1, UBE2V1, CARD10, BCL6, NFKB1, RIPK1, CSNK2B, NFKBIE, CARD11, IKBKB, CARD9, IKBKG
Phagocytosis-Ag pr esentation	39	CTSS, CD1D, CD1A, CD1C, CD1B, CD1E, PSMA1, LAG3, RFX4, PSMB5, PSME1, PSME2, CIITA, CD209, RFX1, IFI30, RFXANK, LILRB5, CD207, XBP1, CD74, PRSS16, HLA-A, HLA-C, HLA-B, HLA-DRA, HLA-DQA1, HLA-DQA2, HLA-DQB2, TAP2, TAP1, PSMB9, HLA-DMB, HLA-DMA, HLA-DOA, HLA-DPA1, HLA-DPB1, TAPBP, NFX1
PI3K/AKT signalin g	37	PIK3CD, FRAP1, PIK3R3, THEM4, AKT3, MAP3K8, PTEN, ILK, RPS6KB2, CCND1, INPPL1, CDKN1B, MDM2, FOXO1A, HSP90AA1, AKT1, TSC2, PDPK1, PIK3R5, MYH4, RPS6KB1, CDC37, PIK3R2, AKT2, LIMS1, INPP5D, CTNNB1, PIK3CB, PIK3CA, EIF4E, GAB1, PIK3R1, CDKN1A, HSP90AB1, NOS3, RHEB, TSC1
ROS/glutathione/ cytotoxic granules	22	PRDX1, NCF2, PRF1, CAT, PRG2, CMA1, GZMH, GZMB, GPX2, ANPEP, NOS2A, GZMM, PRTN3, ELA2, PRDX2, BPI, SOD1, GZMA, GPX3, SOD2, PRDX4, CYBB
TNF superfamily si gnaling	38	TNFRSF4, TNFRSF14, TNFRSF25, TNFRSF9, TNFRSF1B, TNFSF18, TNFSF4, TRAF5, TRAF6, TNFRSF1A, LTBR, TNFRSF7, TNFRSF19, TNFSF11, TNFSF13B, TRAF3, TNFRSF12A, TNFRSF17, TRADD, TNFSF12, TNFRSF13B, TNFRSF11A, TNFSF9, TNFSF7, TNFSF14, TNFAIP6, TNFRSF13C, PTX3, TNIP1, TTRAP, LTA, TNF, TNFAIP3, TNFRSF11B, TNFSF15, TNFSF8, TRAF1, TRAF2
NLRP3-IL1B-IL17 a xis	11	NLRP3, PYCARD, CASP1, IL1B, IL1R1, RORC, CCR6, ATXN1, THY1, CD44, IL17A

Table S2 Agnostic pathway enrichment results of the DMRs identified from the EWAS of effect modification of BMI on adult-onset asthma.

Pathway	P-value	Ratio [a]	Genes [b]
Protein Ubiquitination Pathway	0.000076	0.11	USP35, PSMA7, UBR2, HSPA1A/HSPA1B, CDC23, UBE2W, SKP1, UBE2O, HSPA1L, HSPA4, USP7, UBE2B, STUB1, HSPE1, UCHL5, DNAJC30, BIRC3, UBE2Q1, UBE2M, USP19, DNAJC1, HSPD1, UBE3A, UBE2G2, CBL, HSCB, PSMA5, DNAJC18, PSMD1, HSPB1
ATM Signaling	0.00019	0.15	MAP2K4, PPP2R2A, TRIM28, MAPK9, TDP1, KAT5, PPM1D, USP7, PPP2R1A, BRAT1, RAD17, H2AFX, SMC1B, PPP2R5C, CHEK2
Lysine Degradation V	0.0014	0.60	AASDHPPT, PIPOX, ALDH7A1
Huntington's Disease Signaling	0.0018	0.10	MAP2K4, SGK1, PACSIN1, HSPA1A/HSPA1B, HDAC10, HSPA1L, PRKCZ, HSPA4, CDK5, NTRK1, HDAC7, NCOR1, GNB1L, BET1L, NAPB, MAP2K7, HDAC1, APAF1, MAPK9, HIP1, ATP5F1C, CAPNS1, TAF4, IRS1, NCOR2
Selenocysteine Biosynthesis II (Archaea and Eukaryotes)	0.0026	0.50	SEPHS1, SARS2, SEPSECS
Aldosterone Signaling in Epithelial Cells	0.0035	0.11	SGK1, HSPA1A/HSPA1B, PLCG1, DNAJC1, HSPD1, SLC9A1, PRKCZ, HSPA1L, HSPA4, PIP5K1A, DUSP1, HSCB, IRS1, HSPE1, DNAJC18, DNAJC30, PI4KA, HSPB1
Oleate Biosynthesis II (Animals)	0.0038	0.31	SCD, UFSP2, FADS2, FADS1
Mitochondrial Dysfunction	0.0043	0.11	MAP2K4, NDUFV1, COX4I2, NDUFS7, ACO2, MAPK9, ATP5MG, NDUFB1, NDUFA13, UQCRB, VPS9D1, ATP5F1C, APH1A, NDUFA6, NDUFS6, ATP5MF, GPX4, TXNRD2
HIPPO signaling	0.0062	0.13	YWHAQ, PPP2R1A, YWHAH, PPP2R2A, PPP1R7, SMAD3, NF2, PPP1R14A, PPP2R5C, SKP1, PRKCZ
Estrogen Receptor Signaling	0.0079	0.11	TAF9, MED23, GTF2F2, TAF5L, ERCC2, GTF2A1, TAF4, MED15, ERCC3, MED21, SPEN, TAF3, NCOR1, NCOR2
Thiosulfate Disproportionation III (Rhodanese)	0.0081	0.67	MPST, TST
Cell Cycle: G1/S Checkpoint Regulation	0.0085	0.13	MYC, E2F4, MAX, TGFB1, SMAD3, HDAC7, HDAC1, HDAC10, SKP1
Cell Cycle Control of Chromosomal Replication	0.0089	0.14	MCM5, CDK13, CDK5, CDK11B, ORC6, DNA2, POLA2, CHEK2
IL-1 Signaling	0.0093	0.12	ECSIT, MAP2K4, ADCY9, MAP2K7, TOLLIP, GNAS, GNA12, MAP3K7, MAPK9, GNB1L, ADCY7
Phosphatidylglycerol Biosynthesis II (Non-plastidic)	0.011	0.19	AGPAT5, AGPAT1, MBOAT1, PGS1, MBOAT7
Myc Mediated Apoptosis Signaling	0.011	0.13	FADD, MAP2K4, MYC, YWHAQ, YWHAH, IRS1, APAF1, MAPK9, PRKCZ

[a] Ratio of the number of genes in the DMR to the number of genes in the pathway. [b] Genes overlapping between the DMR and the pathway.

Table S2 (cont.) Agnostic pathway enrichment results of the DMRs identified from the EWAS of effect modification of BMI on adult-onset asthma.

Pathway	P-value	Ratio [a]	Genes [b]
Adipogenesis pathway	0.012	0.10	SAP18, LEP, SMAD3, HDAC1, HDAC10, ERCC2, CDK5, TGFB1, ERCC3, HDAC7, CLOCK, FGFRL1, TBL1XR1, FZD7
D-myo-inositol (1,4,5)-Trisphosphate Biosynthesis	0.013	0.19	PIP5K1A, PI4K2A, PLCG1, PI4K2B, PI4KA
Oxidative Phosphorylation	0.013	0.11	VPS9D1, ATP5F1C, NDUFV1, COX4I2, NDUFS7, NDUFA6, NDUFS6, ATP5MF, ATP5MG, NDUFB1, NDUFA13, UQCRB
PPARα/RXRα Activation	0.015	0.094	MAP2K4, MAP2K7, MED23, GNAS, SMAD3, PLCG1, AIP, ADCY9, TGFB1, IRS1, MAP3K7, CLOCK, NCOR1, SLC27A1, NCOR2, ADCY7, ACVR2A
CDK5 Signaling	0.015	0.11	ADCY9, PPP2R1A, GNAS, CDK5, PPP2R2A, PPP1R7, EGR1, MAPK9, PPP1R14A, PPP2R5C, ADCY7
Spermine and Spermidine Degradation I	0.016	0.50	PAOX, SAT2
Assembly of RNA Polymerase II Complex	0.016	0.14	TAF9, TAF4, ERCC3, TAF5L, TAF3, GTF2A1, ERCC2
Cell Cycle: G2/M DNA Damage Checkpoint Regulation	0.016	0.14	YWHAQ, YWHAH, BORA, SKP1, PRKCZ, CHEK2, PPM1D
tRNA Charging	0.016	0.15	CARS2, HARS, EARS2, HARS2, SARS2, QARS
HGF Signaling	0.019	0.10	MAP2K4, ELF2, PXN, MAP2K7, IRS1, MAP3K7, MAPK9, PLCG1, MAP3K8, STAT3, ELK3, PRKCZ
Pyridoxal 5'-phosphate Salvage Pathway	0.021	0.12	MAP2K4, PNPO, CDK5, SGK1, MAPK9, MAP3K8, HIPK1, ACVR2A
Mitotic Roles of Polo-Like Kinase	0.023	0.12	PLK4, PPP2R1A, PPP2R2A, TGFB1, FBXO5, CDC23, PPP2R5C, CHEK2
ERK5 Signaling	0.023	0.12	MYC, YWHAQ, YWHAH, SGK1, GNA12, NTRK1, MAP3K8, PRKCZ
Lysine Degradation II	0.025	0.40	AASDHPPT, ALDH7A1
Unfolded protein response	0.026	0.13	HSPA4, MAP2K7, SREBF2, HSPA1A/HSPA1B, CANX, NFE2L2, HSPA1L
Cyclins and Cell Cycle Regulation	0.028	0.11	PPP2R1A, E2F4, PPP2R2A, TGFB1, HDAC7, HDAC1, HDAC10, PPP2R5C, SKP1
NRF2-mediated Oxidative Stress Response	0.028	0.088	MAP2K4, MAP2K7, MAPK9, DNAJC1, MAFK, PRKCZ, TXNRD1, BACH1, FTL, KEAP1, IRS1, STIP1, MAP3K7, DNAJC18, GSTO2, FKBP5, NFE2L2
Production of Nitric Oxide and Reactive Oxygen Species in Macrophages	0.030	0.088	MAP2K4, MAP2K7, APOB, PPP2R2A, MAPK9, PLCG1, PPP1R14A, SPI1, PRKCZ, PON1, RHOV, PPP2R1A, PPP1R7, IRS1, MAP3K7, PPP2R5C, MAP3K8
CXCR4 Signaling	0.030	0.091	MAP2K4, PXN, GNAS, MYL2, GNA12, EGR1, MAPK9, PRKCZ, ADCY9, ELMO3, RHOV, IRS1, GNB1L, ELMO1, ADCY7

[a] Ratio of the number of genes in the DMR to the number of genes in the pathway
[b] Genes overlapping between the DMR and the pathway.

Table S2 (cont.) Agnostic pathway enrichment results of the DMRs identified from the EWAS of effect modification of BMI on adult-onset asthma.

Pathway	P-value	Ratio [a]	Genes [b]
Molecular Mechanisms of Cancer	0.030	0.076	MAP2K4, SMAD3, CTNNA1, PRKCZ, MYC, SYNGAP1, CDK5, TGFB1, MAP3K7, BIRC3, CHEK2, CDK13, E2F4, GNAS, GNA12, BMP8B, APAF1, MAPK9, ARHGEF17, FADD, ADCY9, RHOV, CBL, MAX, CDK11B, APH1A, IRS1, ADCY7, WNT1, FZD7
Role of CHK Proteins in Cell Cycle Checkpoint Control	0.030	0.12	PPP2R1A, E2F4, PPP2R2A, RAD17, PPP2R5C, RFC5, CHEK2
Salvage Pathways of Pyrimidine Ribonucleotides	0.032	0.10	MAP2K4, CDK5, SGK1, MAPK9, AK4, UCK1, CMPK1, MAP3K8, HIPK1, ACVR2A
STAT3 Pathway	0.032	0.10	MAP2K4, MYC, SOCS1, TGFB1, NTRK1, MAPK9, FGFRL1, IL27RA, STAT3, NDUFA13
Telomerase Signaling	0.033	0.099	MYC, ELF2, PPP2R1A, PPP2R2A, IRS1, HDAC7, HDAC1, TERT, HDAC10, PPP2R5C, ELK3
CDP-diacylglycerol Biosynthesis I	0.035	0.17	AGPAT5, AGPAT1, MBOAT1, MBOAT7
ERK/MAPK Signaling	0.037	0.085	PXN, YWHAH, PPP2R2A, SRF, PLCG1, PPP1R14A, STAT3, YWHAQ, MYC, PPP2R1A, ELF2, DUSP1, PPP1R7, IRS1, PPP2R5C, ELK3, HSPB1
Glucocorticoid Receptor Signaling	0.038	0.077	CD247, MAP2K4, YWHAH, GTF2F2, SGK1, SMAD3, HSPA1A/HSPA1B, GTF2A1, HSPA1L, HSPA4, TGFB1, MAP3K7, TAF3, NCOR1, FKBP5, TAF9, MAP2K7, TAF5L, MAPK9, STAT3, ERCC2, TAF4, DUSP1, IRS1, ERCC3, NCOR2
TGF-β Signaling	0.041	0.10	MAP2K4, TGFB1, RNF111, SMAD3, HDAC1, SKI, MAP3K7, MAPK9, ACVR2A
Role of JAK family kinases in IL-6-type Cytokine Signaling	0.041	0.16	MAP2K4, SOCS1, MAPK9, STAT3
Induction of Apoptosis by HIV1	0.042	0.11	FADD, MAP2K4, MAP2K7, APAF1, MAPK9, SLC25A3, BIRC3
Breast Cancer Regulation by Stathmin1	0.046	0.083	E2F4, GNAS, CAMK4, PPP2R2A, PPP1R14A, ARHGEF17, TUBB, PRKCZ, ADCY9, PPP2R1A, PPP1R7, IRS1, UHMK1, TUBA1C, PPP2R5C, GNB1L, ADCY7
RAR Activation	0.046	0.084	MAP2K4, SMAD3, MAPK9, ERCC2, PRKCZ, PTEN, ADCY9, TAF4, TGFB1, DUSP1, ERCC3, NCOR1, NCOR2, CSNK2B, CRABP2, ADCY7
Glycolysis I	0.047	0.15	ENO1, PGAM1, PKM, ALDOA
PDGF Signaling	0.049	0.10	MAP2K4, MYC, PDGFA, IRS1, SPHK2, SRF, PLCG1, CSNK2B, STAT3
Thioredoxin Pathway	0.049	0.29	TXNRD2, TXNRD1

[a] Ratio of the number of genes in the DMR to the number of genes in the pathway
[b] Genes overlapping between the DMR and the pathway.

Table S3. Sex difference in BMI effect on adult-onset asthma.

		#Cases	#Controls	Coefficient for BMI [95% CI]	Coefficient for BMI: Female [95% CI]
**All participants** [a]		61	146	0.44 [0.09, 0.79]	-
**Stratification analysis** [b]	**Female**	43	82	0.39 [-0.05, 0.83]	-
	**Male**	18	64	0.51 [-0.13, 1.15]	-
**Interaction analysis** [c]		61	146	0.70 [0.06, 1.35]	-0.37 [-1.13, 0.38]

All participants are non-smoking for at least 10 years and non-atopic
[a] Logistic regression of adult-onset asthma on BMI adjusted for sex, age, education level, study area, and pack-years of cigarettes smoked in life
[b] Logistic regression of adult-onset asthma on BMI adjusted for age, education level, study area, and pack-years of cigarettes smoked in life, stratified by sex
[c] Logistic regression of adult-onset asthma on BMI, sex, and the interaction between BMI and sex, after adjustment for age, education level, study area, and pack-years of cigarettes smoked in life.

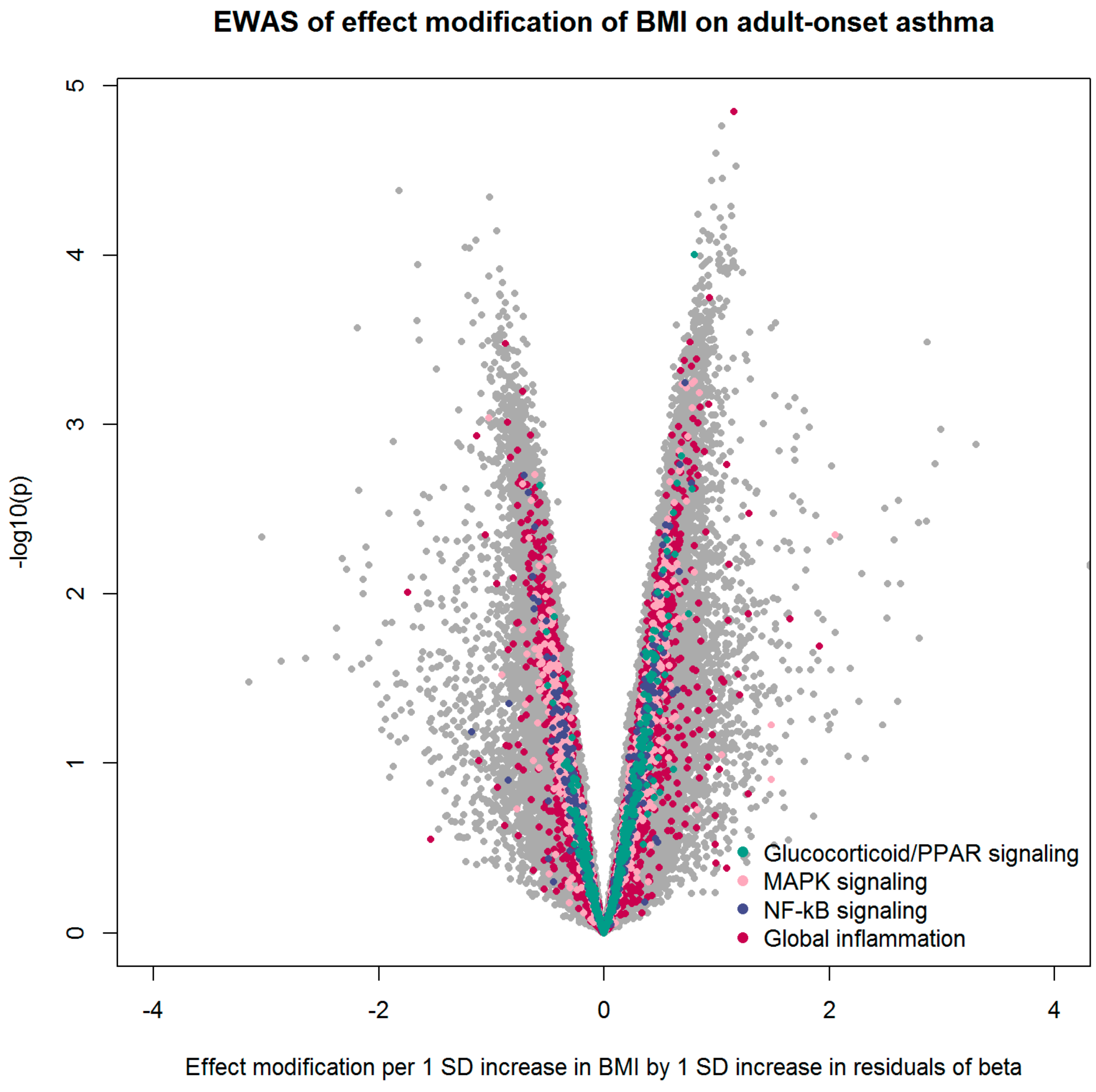

Figure S1. Volcano plot from the EWAS on effect modification by DNA methylation of the BMI-adult-onset asthma association, further adjusted for physical activity.

The EWAS fitted logistic regression models of adult-onset asthma on BMI, residuals of DNA methylation at a single CpG site, and their multiplicative interaction, upon adjustment for age, sex, education level, study area, pack-years of cigarettes smoked in life, physical activity, bench time, and white blood cell composition estimates for B cells, CD4 T cells, CD8 T cells, natural killer cells, monocytes, and eosinophils. The CpGs assigned to the pathway enriched with $p < 0.05$ are highlighted in colors.

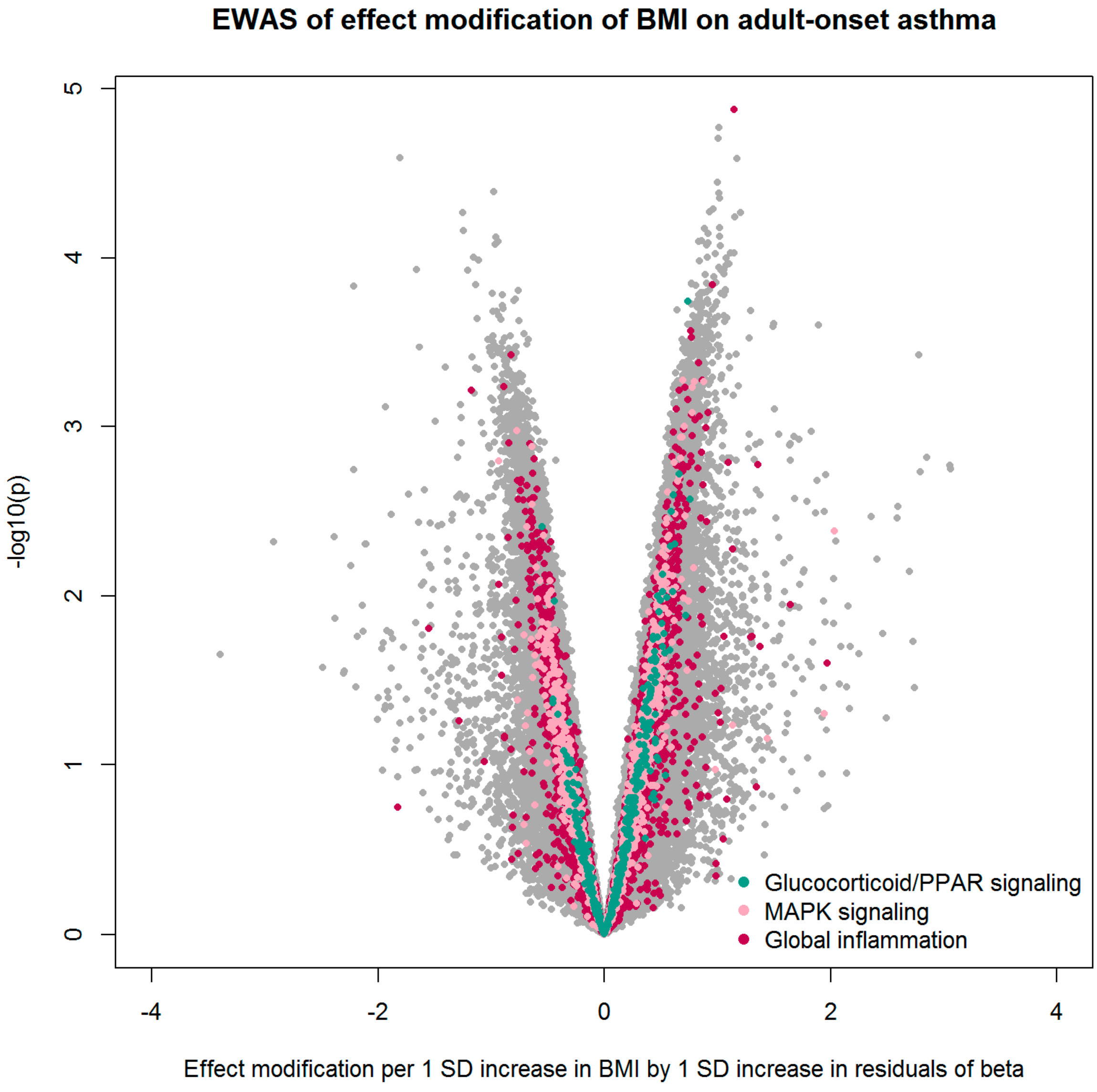

Figure S2. Volcano plot from the EWAS on effect modification by DNA methylation of the BMI- adult-onset asthma association, further adjusted for neutrophil estimates.

The EWAS fitted logistic regression models of adult-onset asthma on BMI, residuals of DNA methylation at a single CpG site, and their multiplicative interaction, upon adjustment for age, sex, education level, study area, pack-years of cigarettes smoked in life, bench time, and white blood cell composition estimates for B cells, CD4 T cells, CD8 T cells, natural killer cells, monocytes, eosinophils, and neutrophils.
The CpGs assigned to the pathway enriched with $p < 0.05$ are highlighted in colors.

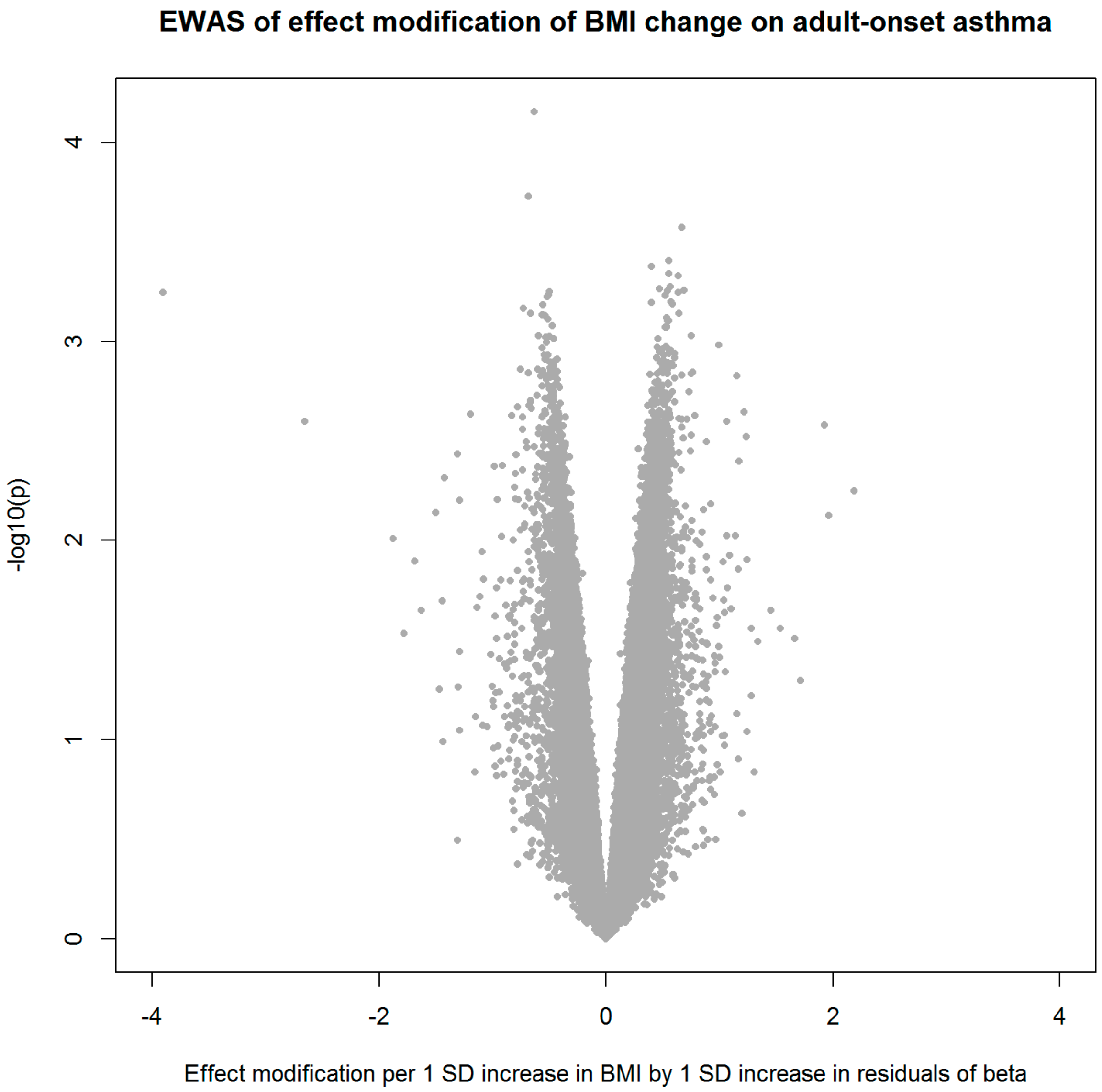

Figure S3. Volcano plot from the EWAS on effect modification by DNA methylation of the BMI change-adult-onset asthma association, further adjusted for physical activity.

The EWAS fitted logistic regression models of adult-onset asthma on BMI change, residuals of DNA methylation at a single CpG site, and their multiplicative interaction, upon adjustment for age, sex, education level, study area, pack-years of cigarettes smoked in life, physical activity, bench time, and white blood cell composition estimates for B cells, CD4 T cells, CD8 T cells, natural killer cells, monocytes, and eosinophils.

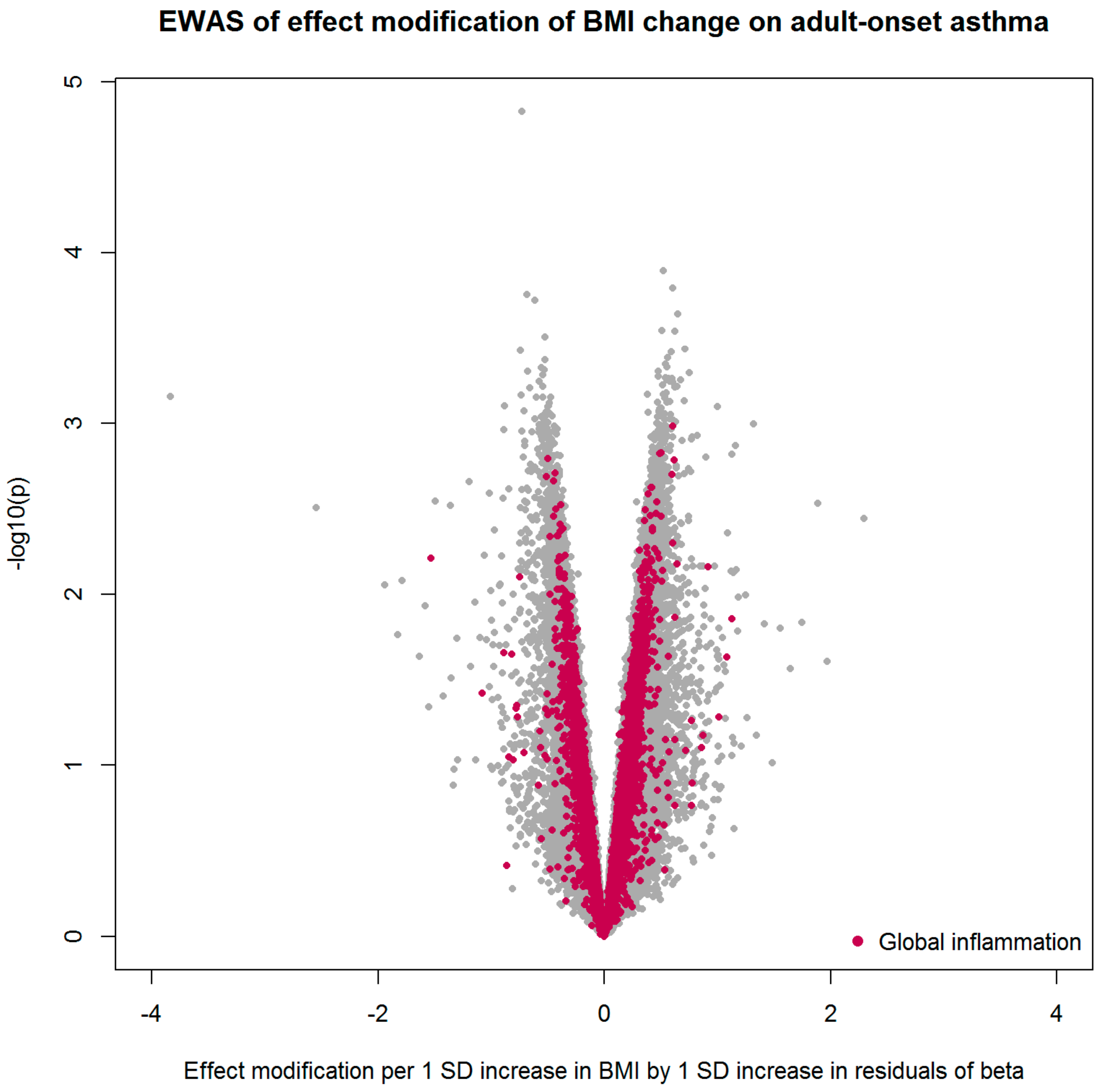

Figure S4. Volcano plot from the EWAS on effect modification by DNA methylation of the BMI change-adult-onset asthma association, further adjusted for neutrophil estimates.

The EWAS fitted logistic regression models of adult-onset asthma on BMI change, residuals of DNA methylation at a single CpG site, and their multiplicative interaction, upon adjustment for age, sex, education level, study area, pack-years of cigarettes smoked in life, bench time, and white blood cell composition estimates for B cells, CD4 T cells, CD8 T cells, natural killer cells, monocytes, eosinophils, and neutrophils. The CpGs assigned to the pathway enriched with $p < 0.05$ are highlighted in colors.

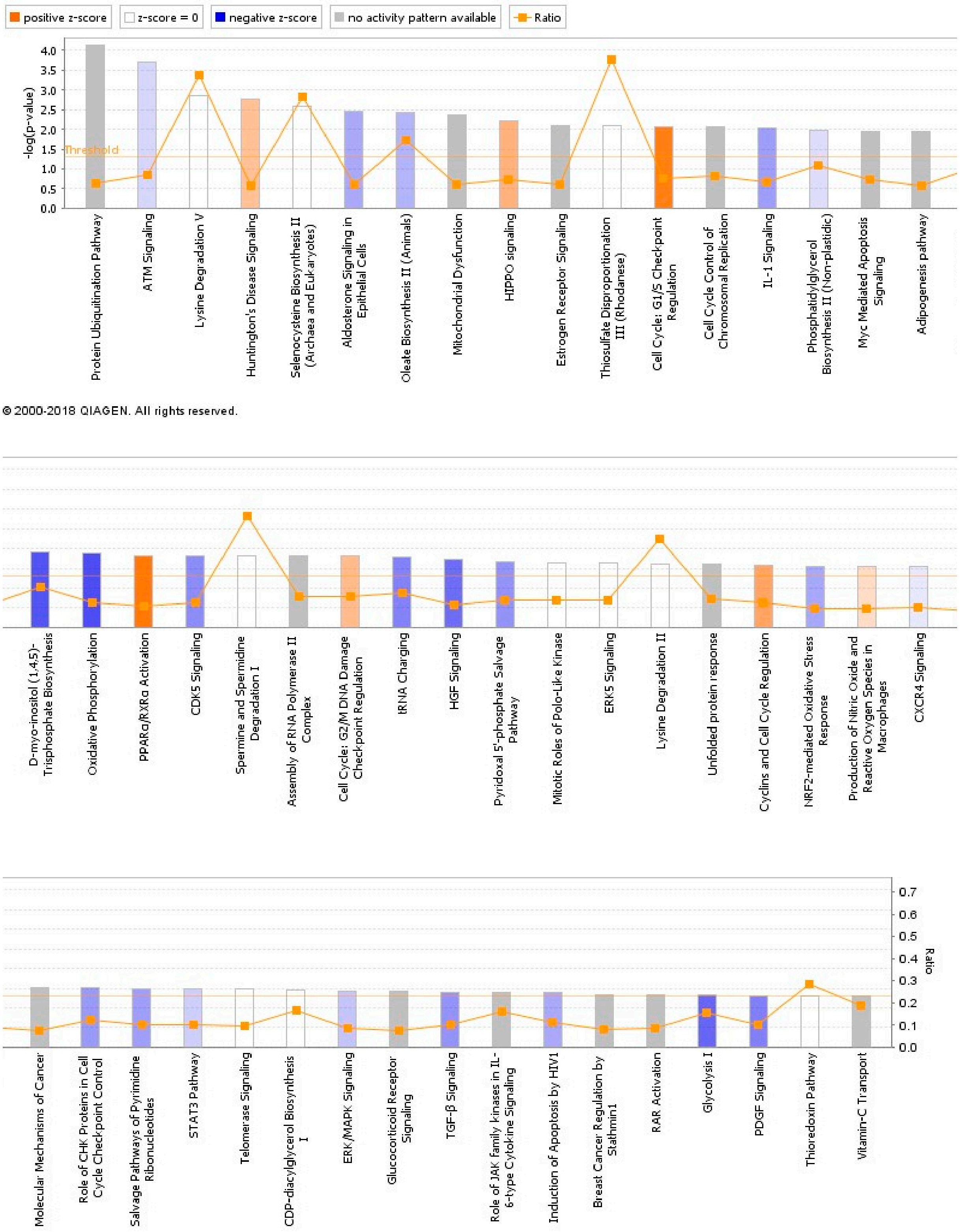

Figure S5. Agnostic pathway enrichment results of the DMRs identified from the EWAS on effect modification of the BMI-adult-onset asthma association.
